# Tourette syndrome research highlights from 2024

**DOI:** 10.12688/f1000research.164800.3

**Published:** 2025-11-07

**Authors:** Andreas Hartmann, Per Andrén, Cyril Atkinson-Clement, Virginie Czernecki, Cécile Delorme, Simon Morand-Beaulieu, Nanette Mol Debes, Kirsten Müller-Vahl, Peristera Paschou, Natalia Szejko, Apostolia Topaloudi, Kevin J. Black

**Affiliations:** 1 Department of Neurology, Pitié-Salpêtrière Hospital, Assistance Publique - Hopitaux de Paris, Paris, 75013, France; 2 Department of Child and Adolescent Psychiatry, Skåne University Hospital, Lund, Sweden; 3 Unit for Child and Adolescent Psychiatry, Department of Clinical Sciences, Lund University, Lund, Sweden; 4 School of Medicine, University of Nottingham, Nottingham, England, UK; 5 Department of Psychology, McGill University, Montreal, Canada; 6 Herlev University Hospital, Department of Child Neurology, University of Copenhagen, Copenhagen, Denmark; 7 Department of Psychiatry, Hanover Medical School, Hannover, Germany; 8 College of Science, Purdue University, West Lafayette, Indiana, USA; 9 Department of Bioethics, Medical University of Warsaw, Warsaw, Poland; 10 Department of Psychiatry, Washington University in St Louis, St. Louis, Missouri, USA

**Keywords:** Tics, Tourette syndrome, annual review

## Abstract

We summarize research reports from 2024 relevant to Tourette syndrome, which the authors consider the most important or interesting. This working draft aims to submit this content for publication around the beginning of 2025 in the yearly Tourette Syndrome Research Highlights series on F1000Research. The authors welcome article suggestions and thoughtful feedback from readers, who can add a comment by clicking on the rectangular comment box icon to the left of the LOG IN link at the top of this page. For private comments, you can reach us by email ( andreas.hartmann@aphp.fr or kevin@wustl.edu).

## Introduction

This article aims to disseminate scientific progress on Tourette Syndrome (TS) that appeared in the year 2024, summarizing research reports the authors judged as important or interesting.

## Methods

We searched PubMed using the search strategy (“Tic Disorders”[MeSH] OR Tourette) NOT ((Tourette[AU] OR Tourette[COIS]) NOT (“Tic Disorders”[MeSH] OR Tourette [tiab])) AND 2024 [PDAT] NOT 1800:2023[PDAT]. On 16 January 2025, 275 citations were retrieved. On the same date, a search of PubMed Central for (“tic disorders”[mesh] OR Tourette *[ti] OR tourette*[ab] OR Tourette*[kwd] OR tourettism[tw]) AND 2024[dp] NOT 1800:2023[dp] returned 264 citations. All these are available in the NLM article section. Eliminating duplicates resulted in 410 unique citations. Colleagues also recommended articles, and selected medical conferences were attended. We selected materials for this review subjectively, guided by our judgment of possible future impacts on the field.

## Results

### Phenomenology and natural history

**Definition and phenomenology**

An analysis of a large TS genetics database examined possible sex differences in people with persistent tic disorders ( Dy-Hollins et al. 2024a). Girls were diagnosed later and less often, but the symptoms started only slightly later (0.5-1.0 years) and were of similar severity. Obsessive-compulsive disorder (OCD) was more common in females and attention deficit hyperactivity disorder (ADHD) was more common in males. Sex differences in patients with tic disorders have also been explored by Gagnon et al. (2024). Females were characterized by lower functional inflexibility (i.e., difficulty switching strategies, adapting to new rules, or adjusting to unexpected changes in environment or routine), worse overall functional planning effectiveness, and higher impairment in psychological well-being subscales compared to males. In addition, girls were characterized by worse quality of life. Conversely, males had more explosive outbursts, more hyperactivity, and experienced more difficulties with self-concept.

Axial tics, that is, tics involving the muscles of the neck, shoulders, and trunk, were observed in 75% of the video recordings of 196 patients with tic disorders ( Baizabal-Carvallo and Jankovic 2024). A greater severity of tics was associated with axial tics in the multivariate regression analysis. Head/neck tics were associated with simple phonic tics, whereas shoulder and trunk tics associated with complex motor tics. The complications observed were neck pain, sleep disturbances, breathing difficulties, and radiculopathy.

Green et al. provided a comprehensive review of pain in TS based on an analysis of 116 articles ( Green et al. 2025). Pain is reported by 47%-60% of individuals with TS, is more prevalent among TS patients than in the general population, and negatively impacts quality of life. A classification system was proposed: tic-related immediate pain, tic-related delayed injury/pain, suppression-related pain, premonitory urge-related pain, and associated primary pain syndromes. In addition, the authors noted that most TS clinical rating scales and outcome measures used in therapeutic studies do not incorporate sufficient information regarding pain. Furthermore, therapies known to improve pain in non-TS conditions that are also reported to improve tics (i.e., botulinum toxin and cannabis/cannabinoids) have not been investigated for their effects on pain in patients with TS media.

A thoughtful commentary on the criteria for primary tic disorders was published by Sarchioto et al., which will hopefully inform future DSM classifications, possibly abolishing somewhat arbitrary distinctions between motor and phonic tics, and also lowering the maximum age of tic onset for diagnosis ( Sarchioto et al. 2024).

Tics and stereotypies frequently coexist in the same patient, and distinguishing them accurately has significant treatment implications. Cavanna et al. provide a systematic literature review that clarifies the diagnosis and management of tics and stereotypies and will guide future research efforts on this important topic ( Cavanna et al. 2024).

Myers et al. found that coprophenomena were reported in 10.1 % of the 169 participants, who were recruited through a multicenter cross-sectional study design ( Myers et al. 2025). Participants with coprophenomena had more severe tics and lower scores for global functioning, family functioning, and quality of life than did participants without coprophenomena.

**Assessment and quantification of tics**

Szejko et al. developed and validated a new scale for the assessment of self-injurious behaviors in tics, the Self-injurious Behavior Scale for Tic Disorders (SIBS-T), and investigated the clinical phenomenology and correlations of self-injurious behavior (SIB). The SIBS-T shows good overall internal consistency. The EFA confirmed a single factor underlying the SIBS-T. SIB was reported in 83.7% of patients, with the most frequent phenotype being beating/pushing/throwing. Patients with SIB had significantly higher tic severity, higher severity of psychiatric comorbidities including obsessive-compulsive symptoms (OCS), attention deficit/hyperactivity disorder (ADHD), and anxiety, these comorbid conditions having detrimental effects on the quality of life of their own, in addition to SIB ( Szejko et al. 2024).

A group from Taiwan ( Zhang et al. 2025) developed and validated the Care Needs Scale for Parents of Children with TS (CNS-PCTS). This instrument was designed to assess the multidimensional needs of parents, including emotional support, informational needs, and practical aspects of caregiving. The scale demonstrated excellent content and discriminant validity, and showed significant correlations with the Pittsburgh Sleep Quality Index, supporting its construct validity in relation to caregiver well-being.

The psychometric properties of the Chinese language and culture of the Motor Tic, Obsessions and Compulsions, Vocal tic Evaluation Survey (MOVES) were investigated by Zhang et al. (2024). The scale showed strong convergent, discriminant, and criterion-related validity, and was significantly correlated with other established TS scales.

Schappert et al. (2024) evaluated an automated video-based approach to diagnose TS in a cohort of 42 adult participants, analyzing a total of 107 videos. Using automated classification of tic frequency and temporal clustering, the system achieved a balanced accuracy of 87.9% for tic quantity and 90.2% for tic clusters. When approximately 25% of the lower-confidence cases were reviewed by experts, the overall diagnostic accuracy exceeded 95% ( Schappert et al. 2024).

**Prognosis and natural history**

Fernández de la Cruz et al. examined mortality in a matched and sibling cohort study in which all individuals diagnosed with chronic tic disorder or TS from the Swedish National Patient Register were matched to healthy controls from the general population (1:10) and compared to their siblings without tics. Individuals with chronic tic disorders or TS have been shown to have an increased risk of mortality due to both natural and unnatural causes ( Fernández et al. 2024).

Two reports provided the first large prospective follow-up study on provisional tic disorder (PTD). Liu et al. studied prognostic factors in children with PTD. After 1 year of follow-up, clinical remission was observed in 30% of cases. Persistence of tics (non-remission) was associated with longer disease duration (>3 months), greater tic severity, and comorbid behavioral symptoms ( Liu et al. 2024). A report from the US used a more intensive assessment 12 months after tic onset ( Grossen et al. 2024). At that visit, tics were observed directly during 70% of standard clinical encounters. However, when the children were additionally monitored on video while alone in the room, tics were present in all but one of 79 cases. This result is consistent with prior clinical experience, where the absence of tics during routine examination is common. The authors concluded that PTD is much more similar to TS than different, and they argue that the traditional but arbitrary separation of provisional from chronic tic disorders should be reconsidered.

A study from the US ( Barber et al. 2024a) investigated contextual triggers for tics across different life periods. Overall, tics worsened during and after school during school years and aggravated in the evening during the college/work years. In addition, similar to previous reports, stress and anxiety have been reported to be consistent worsening factors.

Bootes et al. (2024) investigated predictors of functional impairment and self-concept in children and adolescents with persistent tic disorders. They found that poorer self-concept (a more negative view of oneself) was associated with comorbid anxiety and depression, greater tic severity and complexity, and lower quality of life.

**Sensory phenomena and premonitory urge**

Prato et al. investigated sensory phenomena in children with TS and autism spectrum disorder (ASD) using the University of Sao Paulo’s Sensory Phenomena Scale (USP-SPS) ( Prato et al. 2024). They found that sensory phenomena were more prevalent and severe in the ASD group compared with the TS group, except for sound-related “just-right” perceptions and the sensation of energy to be released. On average, ASD participants scored higher than both TS patients and healthy controls across all USP-SPS severity domains. Greater severity of sensory phenomena was associated with more severe obsessive-compulsive and anxiety symptoms; in the ASD group, it was also positively correlated with ADOS total scores and negatively correlated with IQ.

Kurvits et al. investigated rapid compensation for «noisy» voluntary movements in individuals with tic disorders, based on the premise that tics and premonitory movements may reflect increased sensorimotor noise (random variability in the neural signals controlling movement). They found that patients with tics produced more variable voluntary movements than controls, but were able to rapidly adjust their performance according to current task demands ( Kurvits et al. 2024).

**Comorbidities**

Forlim et al. (2024) conducted a symptom network analysis in a large sample of children and adults with chronic tic disorders. In the overall network, complex motor and vocal tics, as well as behaviors such as touching people or objects, emerged as central (highly connected) symptoms ( Forlim et al. 2024). In children, attention-deficit/hyperactivity disorder (ADHD) symptoms were among the most influential nodes, whereas in adults, obsessive-compulsive disorder (OCD) symptoms were more central. Self-injurious behaviors were peripheral in children but constituted a core feature in the adult network. The analysis also revealed strong interconnections between complex tics, echolalia, and echopraxia.

Jensen et al. investigated the correlation between comorbidities and tic variability in children with tics. As demonstrated in other studies, patients with comorbidities had a higher tic severity and impairment than those in the tic-only group ( Jensen and Debes 2024). Specifically, this study showed significantly higher simple photonic tic scores in the CTD+OCD group than in the CTD-only group [CTD = chronic tic disorder].

Zoccante et al. examined the prevalence of connective tissue-related conditions in individuals with TS, ASD, and ADHD ( Zoccante et al. 2024). Compared with controls, flat feet and hypersensitivity were more common in patients with TS, which could be caused by shared etiological pathways.

The frequency of co-occurring somatic diseases was examined retrospectively among outpatients with tic disorders who visited Beijing Children´s Hospital between January 1, 2018, and December 31, 2022 ( Yu et al. 2024). A total of 523,462 visits were included in this study. The most commonly diagnosed diseases are upper respiratory tract infections, conjunctivitis, dyspepsia, and dermatitis.

Cheng et al. conducted a retrospective study of 182 newly diagnosed patients with tic disorder who were screened for comorbidity using the Mini International Neuropsychiatric Interview for Children and Adolescents 5.0 ( Cheng et al. 2024a). Several factors are associated with the presence of comorbidities. The presence of comorbidities was associated with more severe tics, complex vocal tics, delayed diagnosis, single-parent households (compared to two-parent households), and a higher frequency of psychological factors (e.g., anxiety and anger). These factors are important to keep in mind when targeting support to patients and their families.

Temperament traits in patients with pediatric OCD in relation to TS and ADHD have been investigated by Cheng et al. ( Cheng et al. 2024b). The complex phenotype, being a combination of all three disorders, is characterized by higher novelty seeking and lower persistence. Conversely, harm avoidance increased in all groups compared with controls. Other associations included contamination and washing symptoms related to higher novelty seeking, whereas counting and ordering were related to lower novelty seeking. Finally, harm avoidance was increased in aggressive, somatic, and checking symptoms in OCD only, while persistence increased with repeated and counting symptoms in the comorbid groups (OCD+ADHD or TS, OCD+ADHD+TS).

Katz et al. published an interesting review on the intersection between repetitive behaviors in TS, OCD, and autism ( Katz et al. 2025). They concluded that, at least in some cases, TS and OCD belong to the same spectrum that should be named “Tourettic OCD,” a phenotype at the intersection of tics and OCD.

Sensory over-responsivity was examined in 26 adult patients with chronic tic disorders and 31 neurotypical adults. Since many adults with tic disorders experience sensory over-responsivity, it was hypothesized that sensory gating impairment could be the cause, but the results obtained could not support this hypothesis ( Isaacs et al. 2024a).

The presence of non-obscene socially inappropriate behaviors (NOSIBs) was examined in a cohort of 365 participants with TS with an age range between 4-64 years and mean age 14.4 years (76.2% children) ( Grycz and Janik 2024). The prevalence of NOSIBs was 23.6% with a mean age of onset of 6.6 ± 4.1 years and on average 1.4 ± 3.7 years after the onset of tics. The presence of NOSIB was associated with the severity of tics and the presence of ADHD, oppositional defiant disorders, ASD, and anxiety disorders. This might indicate that a more severe disease course is a risk factor for NOSIB development.

Macro- and micronutrient intake and food selectivity in 43 children with TS and 38 children without TS were examined by Smith et al. (2024). Children with TS consumed fewer portions of fruit and vegetables and had lower protein and higher starch intake than healthy controls. The intakes of other macro- and micronutrients were similar between the two groups. 58% Of children included in this study had abnormal body mass index (BMI) (underweight, 24.2%; overweight, 27.3%; obese, 6.1%). In addition, children with tics consumed less fruits and vegetables and nutrients rich in vitamins B _3_, B _6_ and C, selenium, and phosphorus compared to children without TS. These findings suggest that children with TS may have a tendency toward a more nutritionally unbalanced diet. As poor food choices have been linked to adverse mental and physical consequences later in life, it is important for professionals to be aware of the nutritional intake of patients with TS.

Esmaeilzadeh et al. (2024) conducted a case–control study in Iran comparing three groups: patients with both tic disorders and allergic rhinitis (AR), patients with tic disorders without AR, and patients with AR without tics ( Esmaeilzadeh et al. 2024). They found that vocal tics were more frequent among patients with both tics and AR, and these individuals also experienced more days per week with AR symptoms. The most common tic diagnosis in the sample was provisional tic disorder. These findings suggest a possible interaction between allergic symptoms and tic expression, highlighting the need to consider comorbid allergic conditions in the assessment and management of tic disorders.

Li et al. examined the role of sleep in a cohort of 150 children and adolescents (age 4-14 years) with tic disorders and found significant correlations between the severity of tics, sleep (measured with Children’s Sleep Habits Questionnaire), and quality of life) ( Li et al. 2024). Sleep, especially bedtime resistance, mediates the relationship between tic severity and quality of life. Bedtime regularity and sleep sufficiency have been investigated by researchers from California ( Swisher et al. 2024). Children with TS had poorer bedtime regularity, but not sleep sufficiency, than matched controls. Importantly, anxiety and two or more hours of screen time per day were related to worse bedtime regularity. Conversely, autism is associated with a lower likelihood of insufficient sleep. The coexistence of depression was associated with a higher likelihood of insufficient sleep. Keenan et al. (2024). 85% of children with TS scored in the clinical range for a sleep disorders. A higher tic severity is associated with sleep and executive problems.

The inter-rater (dis) agreement between self-, mother, and father regarding quality of life was investigated by Jalenques et al. (2024). Agreement between mother, father proxy reports, and TS adolescents’ self-reports of quality of life varied from poor to good, without significant differences from the control group. In the TS group, mothers and fathers underestimated adolescents’ quality of life in the Psychological ‘well-being’ subscales, mothers underestimated it in the Physical ‘well-being’ subscales, and controls overestimated adolescents’ quality of life in all of these subscales. Larger mother-adolescent discrepancies for the “psychological well-being” and “physical well-being” subscales were associated with internalizing symptoms.

A study from Ohio State University ( Morgan et al. 2024) has shown that administration of electronic quality of life instruments in patients with tics significantly improved compliance and survey completion from 51.9% to 91.6%, which should guide further efforts in quality of life studies for individuals with TS.

**Functional tic-like behaviors**

The Calgary team published two noteworthy papers on functional tic-like behaviors (FTLB) in 2024. First, they tested the specificity of phenomenological criteria for FTLB according to the European Society for the Study of Tourette Syndrome (ESSTS) criteria published in 2023 ( Pringsheim et al. 2023), in 156 children recruited within the Calgary registry ( Nilles et al. 2024a). There was high specificity (94.2%) for the age at onset criterion (≥12 years) and for having at least two complex motor and one complex phonic behavior at first visit (96.2%). Some of the complex motor tics had a lower specificity. The specificity of the FTLB diagnostic criterion for having more complex tics than simple tics was 89.7%. Overall, these findings support the use of ESSTS criteria for FTLBs in clinical practice. Second, they attempted to describe the phenomenology of FTLB in youth and assess the movements and vocalizations most suggestive of the diagnosis in 236 youths (195 with primary tics, 41 with FTLB) from the Calgary tic registry ( Nilles et al. 2024b). In a bivariate model, FTLB was most associated with copropraxia, saying words, coprolalia, popping, whistling, simple head movements, and self-injurious behaviors. In the multivariable model, the FTLB was still associated with words and simple head movements.

The prevalence of a subtype of FTLB, mass social media-induced illness presenting with Tourette-like behavior (MSMI-FTLB), was explored by authors from the Hannover Medical School ( Hartung et al. 2024). Probable MSMI-FTB was found in 33 individuals (mean age at onset: 30.5 years, n = 8 females). Depending on selection criteria, MSMI-FTLB was found to have a prevalence of 0.17% - 0.36% showing that it is an important public health problem.

A study from Harvard University ( Tomczak et al. 2024), evaluated the clinical phenomenology and prognosis of 56 patients with functional tic-like behaviors. Similar to other studies, there was a high predominance of female sex (93%) and prevalence of depression (71%). Interestingly, 45% of individuals were gender diverse. Again, in line with previous research, the majority of patients (79%) improved in follow-up, independent of comorbid diagnosis or treatment. The authors also compared tic-specific treatments to other treatment modalities and found no disparities in clinical response.

Berg et al. examined social and psychological factors associated with FTLB ( Berg et al. 2024a). The burden of psychiatric comorbidities, especially depression and panic disorders, was significantly higher in individuals with FTLB than in those with primary tics or controls. In addition, borderline personality disorder (BPD), sleeping problems, agoraphobia, social anxiety disorder, and generalized anxiety disorder were more frequent in the FTLB group. Vulnerable attachment scores, social phobia, and social interaction anxiety were higher in the FTLB group than in the control group, but not in individuals with TS. Surprisingly, distress tolerance, resilient coping, suggestibility, hours spent on social media, and exposure to tic and TS content did not differ between the groups. In addition, the COVID-19 pandemic had a worse impact on individuals with FTLB than on individuals with TS or controls. From a social perspective, loneliness, difficulty in affording housing, and food were also more frequent in FTLB. The prevalence of gender minority individuals was also higher among individuals with FTLB. The same group explored the impairment and health impacts of the FTLB ( Berg et al. 2024b). In this study, 35 individuals with FTLB, 22 with TS, and 25 healthy controls were compared. Again, a significantly higher proportion of individuals with FTLB were identified as belonging to the gender minority group. Compared to controls, the FTLB group was characterized by a lower quality of life, greater disability, loneliness, social phobia, anxiety, depression, and suicidality. Similarly, individuals with FTLB experience more school/work absenteeism.

Armstrong-Javors et al. demonstrated an increase in FTLB among gender-minority children after the COVID-19 pandemic ( Armstrong-Javors et al. 2024). The prevalence of functional tic presentations in youth rose 8.6-fold from the pre- to post-pandemic levels, whereas the prevalence of developmental tic presentations pre- and post-pandemic remained stable. The prevalence of sex- and gender- sex-minority in FTLB was estimated to be 37%, which is considerably higher than in the general population.

Ludlow et al. investigated mothers’ experiences with FTLB symptoms in their children ( Ludlow et al. 2024). Semi-structured interviews identified the main themes evolving around the occurrence and development of tics, the severity and intensity of symptoms, the psychological impact on the family, and the need to make recommendations for a clear care pathway. The management of suicidal ideation, self-harm, and physical and emotional trauma was particularly challenging for mothers.

A critical examination of FTLB diagnosis was proposed by underlining the potential relevance of circular reasoning owing to the lack of clinical benchmarks ( Andersen et al. 2024).

**Epidemiology**

A nationwide, population-based survey of children’s health in the USA investigated the prevalence of diagnosed TS in a nationally representative sample of 278,472 children and adolescents (0-17 years), and found a prevalence of 0.23% ( Xiong et al. 2024). TS was defined by a parent/guardian’s affirmative answer to the question: “Has a doctor or other health care provider ever told you that this child has Tourette syndrome?”. A significantly higher prevalence was found among boys (0.35%) than girls (0.11%), and prevalence was lower in non-Hispanic Blacks (0.16%) compared to Hispanics and non-Hispanic Whites (0.22% and 0.28%, respectively). The estimated prevalence of TS has not changed significantly between 2016 and 2022.

### Etiology

**Genetics and epigenetics**

Strom et al. performed the largest TS GWAS meta-analysis to date, including 9,619 cases (4,800 of which were new) and 981,048 controls of European ancestry ( Strom et al. 2025). In a primary GWAS, they identified a genome-wide significant variant of MCHR2-AS1. They were not able to replicate their genome-wide significant hits in an independent sample of 885 TS cases and 310,367 controls, but they replicated a hit from a previous large TS GWAS (rs2453763) reported by Tsetsos et al. (2024). Post GWAS gene-based analyses revealed three additional significant genes: BCL11B, NDFIP2, and RBM26. Tissue enrichment analyses showed enrichment of TS variants in genes expressed in the cortico-striato-thalamo-cortical circuit and five brain cell types (excitatory and inhibitory telencephalon neurons, inhibitory diencephalon and mesencephalon neurons, and hindbrain and medium spiny neurons).

An additional TS GWAS was performed by Lin et al. (2024) in a non-European population ( Lin et al. 2024). They analyzed 1,007 TS cases and 25,522 ancestry-matched controls from Taiwan and identified a significant genetic locus on chromosome 12q23.2, implicating DRAM1, which is involved in autophagy and apoptosis.

Another study on non-European samples was performed by Lu et al. (2024), who analyzed whole-exome sequencing data from 390 individuals with TS and 372 controls in a Chinese Han population to identify TS risk genes. They identified 14 potential TS susceptibility variants and 10 variants as potential disease-causing variants of TS, all of which are located in known TS candidate genes, such as COL27A1, WWC1, and NRXN1. Lastly, pathway analyses of 354 previously unknown TS genes found enrichment in PI3K-Akt signaling, sphingolipid metabolism, and serotonergic synaptic pathways.

Ko et al. performed a preliminary Epigenome-wide Association Study on 16 individuals with TS and 24 controls from a Korean population, where they explored the differentially methylated regions (DMRs) separately in males and females ( Ko et al. 2024). In male samples (n=28), they identified seven DMRs hypermethylated in individuals with TS and 30 DMRs hypermethylated in controls, while in females (n=12), they found 28 DMRs hypermethylated in individuals with TS and 10 DMRs hypermethylated in controls. Follow-up functional enrichment analyses of the DMRs by CpG revealed hypomethylated patterns in the ligand, receptor, and second signal transductors of the PI3K-Akt and MAPK signaling pathways in males, whereas the opposite patterns were observed in females. Looking into specific genes, the HLA-DRB1 gene was hypomethylated in males, whereas in females, HLA-DQB1, HLA-DPB1, and HLA-DPA1 genes were hypermethylated. In males, they also found that PTPN1, RUNX1, and SLC1A7 were hypermethylated in TS cases, and that SLC1A1 was hypomethylated.

Fichna et al. analyzed structural variants using whole genome sequencing data of 17 multiplex families, including 80 TS cases from Poland, and 102 external controls ( Fichna et al. 2024). Their analyses showed 97 putative pathogenic rare variants (<1% in controls) and 70 putative pathogenic variants shared among affected individuals within one family, but not present in the control group (so-called private variants). Four of these private or rare variants were exonic (LDLRAD4, B2M) or 3’-UTRs (USH2A and ZNF765). Enrichment analyses revealed that these structural variants were primarily found in genes involved in key biological processes such as neurite outgrowth signaling, cell leading-edge organization, and synaptic vesicle endocytosis.

Miller-Fleming et al. performed a phenome-wide association study (PheWAS) to explore which electronic health records (EHRs) were enriched in TD cases (n = 1406) compared to controls (n = 7030) ( Miller-Fleming et al. 2024). Among the detected EHR features were psychiatric disorders such as OCD, ADHD, autism spectrum disorder, anxiety, and neurological traits including extrapyramidal disease, abnormal movement disorders, abnormal movement, and torsion dystonia. They used the identified EHR (n = 69) to construct a phenotype risk score (PheRS) for tic disorders, which they applied to an independent sample of 90,051 individuals and included a clinician-validated TD case set (n = 266). By performing a logistic regression analysis in the independent sample, they found that clinically validated cases had a significantly higher TD PheRS than non-cases.

**Environmental risk factors**

Hu et al. examined the role of family functioning in tic severity and quality of life in 139 children with TS ( Hu et al. 2024a). Dysfunction in family communication played a role in linking the severity of tics to both psychological symptoms and difficulties with physical abilities and daily activities. This effect was even stronger for vocal tics and was affected by sex and ADHD. Evaluating family functioning is thus important for understanding the quality of life of children with TS.

Salehi et al. used data from the National Survey of Children’s Health in the US from 2020 to 2021 and included 91,404 children and adolescents (0-17 years) for the prevalence of tobacco smoke exposure, and 79,182 children and adolescents (3-17 years) for the analysis neurological and psychiatric of comorbidities in order to examine a possible link between tobacco smoke exposure and neuropsychiatric conditions ( Salehi et al. 2024). They found that 36.4% of adolescents with tobacco smoke exposure developed at least one comorbidity, mainly anxiety problems (15.7%), Attention-Deficit Hyperactivity Disorder (ADHD) (15.5%), behavioral and conduct problems (13.7%), and learning disability (12%). Also, and tobacco smoke exposure significantly increased the risk fo developping TS (OR: 4.4, *p* < 0.001). The authors conclude that « Screening for these conditions in youths exposed to tobacco smoke exposure is crucial for early detection and interventions to increase their mental health and well-being. »

Twenty-four patients with obsessive-compulsive tic disorder, an internal classification regrouping patients with chronic tics and OCD, were included in a longitudinal study lasting 15 months in Italy ( Lamanna et al. 2024). Local environmental data and the severity of tics and obsessive-compulsive symptoms were assessed at six time points. Tics were more severe in spring and summer than in winter and autumn, and ambient temperature was positively associated with the severity of tics. This pattern was not observed for OCD symptoms.

A study from China investigated the impact of dietary inflammatory index on tic severity ( Wu et al. 2024). Patients included in this study were divided into stable (n = 177) and recurrent (n = 90) groups, but there were no statistically significant differences in demographics, BMI, and disease duration between the two groups. Overall, there was a positive correlation between dietary inflammation and tic severity, tic recurrence, and inflammatory biomarker levels. The authors concluded that a reasonable reduction in the intake of proinflammatory foods could be beneficial for patients with tics.

### Pathophysiology

**Electrophysiology**

In 2024, Takacs et al. published two papers that investigated statistical learning among adults with TS ( Takacs et al. 2024a, 2024b). Both papers stemmed from the same study, in which 25 adults with TS performed the cued version of the Alternating Serial Reaction Time task. Analyses of task performance revealed that adults with TS showed a higher statistical learning score than the controls, but the groups did not differ in terms of rule-based learning. Statistical learning refers to the process of automatically detecting patterns and probabilistic relationships in data, enabling the system to make predictions and generalize beyond the specific examples encountered during training, often using algorithms that learn from large datasets. In contrast, rule-based learning relies on explicitly defined rules created by human experts, where the system follows fixed, deterministic instructions for decision making and cannot adapt to new patterns unless the rules are manually updated. Nevertheless, the neural representations for statistical and rule-based regularities differed between adults with TS and the controls. These representations in the frontoparietal brain regions were maintained for longer intervals in those with TS for both statistical and rule-based regularities ( Takacs et al. 2024a). In another study, the authors found that the resting-state theta network, which is involved in statistical learning, had more small-world-like properties in adults with TS than in controls ( Takacs et al. 2024b). Small-world-like properties refer to features of networks where most nodes can be reached from every other node by a small number of steps (short average path length), while also maintaining a high degree of clustering (where neighbors of a node are also likely to be connected to each other). Additionally, participants with TS presented with higher theta network segregation induced by learning during the alternating serial Reaction Time task. These two studies highlight the propensity for hyperlearning among individuals with TS, which is associated with distinct neural processes.

Another EEG study focused on taboo words in patients with TS. Coprolalia is a well-known symptom of TS, but the neural correlates underlying the production of taboo words in people with TS are unclear. This event-related potential (ERP) study used a task designed to induce spoonerisms (i.e., swapping of word sounds in a short phrase) to assess the suppression of taboo and non-taboo words in adults with TS and age-matched controls ( Robert et al. 2024). While controls and adults with TS did not differ in terms of the occurrence of spoonerism, frontal ERP activity was larger for taboo words than for non-taboo words. This finding suggests that more cognitive control processes are recruited to inhibit taboo words in TS.

As mentioned in the treatment section (see below), deep brain stimulation (DBS) is an efficient treatment mostly reserved for severe cases in which traditional treatment options (e.g., psychopharmacology and psychological interventions) may not work. However, the electrophysiological effects of these treatments remain poorly understood. An EEG study among adults with TS explored the impact of thalamic DBS on cortical beta activity, an oscillatory frequency band associated with sensorimotor integration ( Schüller et al. 2024). The authors reported that DBS activation increased beta power, primarily due to modulations in the midcingulate cortex, suggesting that thalamic DBS may enhance the cortico-subcortical network function by restoring beta oscillations. DBS surgery also offers a rare opportunity for direct electrophysiological recording of the basal ganglia. Lamothe et al. recorded the electrophysiological activity of the internal globus pallidus (GPi) in patients with TS and dystonia undergoing DBS ( Lamothe et al. 2024). They found a higher firing rate, firing rate within the bursts, pause duration, and interspike interval coefficient of variation in patients with TS than in those with dystonia. However, there was a higher frequency of pauses and bursts, as well as increased oscillatory activity (especially in the theta, alpha, and high beta bands) in dystonia. These features may reflect the brief and phasic nature of tics compared to dystonic movements and reflect important differences in the pathophysiology of TS and dystonia.

Finally, sensory gating remains an important research topic in the neurophysiology. In a study of auditory gating, Isaacs et al. found no significant difference in the P50, N100, and P200 auditory gating ratios between adults with TS and controls ( Isaacs et al. 2024b). Although adults with TS showed enhanced sensory over-responsivity relative to controls, these findings do not suggest that auditory gating may be considered a mechanism of sensory over-responsivity in TS.

**Neuroimaging studies**

Two interesting studies focused on pediatric patients with TS with the aim of identifying MRI biomarkers. Che et al. conducted an impressive study involving 187 children, including 60 healthy controls, 39 with TS, and 88 with tic onset for less than nine months ( Che et al. 2024). They assessed the volume and shape of subcortical nuclei and found abnormalities across all nuclei when comparing healthy participants with those with TS. However, patients with recent tic onset displayed only a few significant differences: a larger hippocampus compared to the healthy group and an enlarged pallidum and thalamus, which were associated with less tic improvement.

Similarly, Luo et al. explored functional and effective connectivity dysfunctions using fMRI in 54 drug-naïve patients with TS compared with 46 healthy participants ( Luo et al. 2024). The authors identified two brain states and found that patients with TS had a higher occurrence of a state characterized by increased connectivity. They highlighted the pivotal role of the Default Mode Network in these states and related it to tic severity.

A study conducted by Zapparoli et al. examined different aspects of tic-related behaviors ( Zapparoli et al. 2024). They used a mental imagery task to identify several phases, including the premonitory urges, tic execution, and inhibition. Notably, the distress experienced during urge imagery activates a network primarily associated with the sensorimotor areas of the brain. In contrast, the relief felt during tic imagery engaged the inferior frontal cortex, insula, and basal ganglia, whereas during tic inhibition was correlated with activity in the superior frontal gyrus. Overall, this study delineated the distinct neural networks involved in different aspects of tic behavior.

Orth et al. investigated the role of the salience network in TS ( Orth et al. 2024). They used seed-based resting-state functional connectivity to focus on the roles of the insula, anterior cingulate cortex, and temporoparietal junction. By comparing 26 adults with TS to 25 healthy controls, they identified over-connectivity in individuals with TS (insula with the central operculum, anterior cingulate cortex with motor areas, temporoparietal junction with prefrontal areas) and one decreased connectivity between the insula and thalamus. Notably, more severe tic symptoms, as measured by the Yale Global Tic Severity Scale (YGTSS), were correlated with lower connectivity between the insula and superior frontal gyrus on both sides. Overall, these findings highlight the relationship between salience processing and tic production in TS.

**Neuropsychology**

Morand-Beaulieu et al. provided an overview of neurocognitive functioning in individuals with TS, highlighting both challenges and strengths across cognitive domains, while considering potential confounding factors ( Morand-Beaulieu et al. 2025). Although a longer clinical history of tics has been associated with negative impacts on cognitive functioning, they suggested that the cognitive deficits observed in individuals with TS are influenced more by comorbid conditions such as ADHD and OCD than by the TS itself. Additionally, they highlighted differences in cognitive performance between children and adults with TS, particularly in executive function, emphasizing the role of compensatory neural mechanisms. These findings underscore the need for further research to disentangle TS-specific cognitive traits from comorbid influences, which could inform more targeted interventions.

Conte et al. examined how comorbidities and disease duration influence cognitive functioning and quality of life (QoL) in children with TS ( Conte et al. 2024). Research conducted on 80 children aged 6–16 years found that ADHD and depression were linked to poorer cognitive performance, while anxiety showed a positive correlation with cognition. A longer TS duration was associated with lower IQ, impaired verbal reasoning, and working memory. Additionally, depression, anxiety, and disease duration significantly affected QoL, emphasizing the need for early assessment and intervention in TS management.

**Animal models**

A reduction in striatal acetylcholine levels has been suggested to contribute to tic genesis. Using CIN-d and D1CT-7 mice, Caddedu et al. tested whether activating M _1_ and/or M _4_ receptors might decrease tic-related behaviors in mouse models of TS ( Cadeddu et al. 2024). Activation of striatal M _4_, but not M _1_, receptors reduced tic-like manifestations in mouse models, suggesting that xanomeline (a selective M _1_/M _4_ receptor agonist) and M _4_ positive allosteric modulators may be novel therapeutic strategies for TS, extending the range of neurotransmitter targets beyond dopamine. The same group also published an interesting review on the role of neuroactive steroids in tic disorders ( Branca and Bortolato 2024).

### Treatment

**General**

Several reports have provided important information for the design of future treatment studies for TS. Wand et al. summarized the pill placebo response rate in patients with tic disorders. They found a pooled effect size of −0.79 ( Wang et al. 2024a). A pooled effect size refers to the combined effect across several studies, summarizing the average outcome for the placebo group. They also noted that 44% of study participants reported adverse events with placebo. Macul et al. reported a similar meta-analysis of 50 randomized controlled trials (RCTs), with an effect size of −0.62, and identified non-US trials, industry support, and the number of centers involved in the study as associated with a greater placebo response ( Macul et al. 2024). However, these factors are also associated with a higher response to active drugs. Consequently, addressing these risk factors may not increase the difference between drug and placebo effects, which is of real interest.

**Psychological interventions**

Current clinical guidelines from both the American Academy of Neurology (AAN) and the European Society for the Study of Tourette Syndrome (ESSTS) endorse behavior therapy (BT) as the preferred first-line treatment for TS/CTD ( Pringsheim et al. 2019; Müller-Vahl et al. 2022). Among the available BT approaches, habit reversal training (HRT) and its expanded protocol, Comprehensive Behavioral Intervention for Tics (CBIT), are supported by the most robust evidence. Another form of BT, Exposure and Response Prevention (ERP), has received less support to date but is particularly favored by many European clinicians and researchers.

Barber et al. published an 11-year follow-up study of a large adult sample (originally *N* = 122, now *N* = 72) who received either CBIT or supportive therapy ( Barber et al. 2024b). This study aimed to investigate participants’ perceived negative effects of using tic management strategies as well as potential predictors of such experiences. While most participants did not report tic worsening or tic substitution, approximately half reported feeling less present when managing their tics. Overall, no significant differences were observed between the two treatment groups in terms of negative effects. The authors concluded that these findings may help reduce misconceptions regarding BT for TS/CTD and enhance its acceptability among users.

In recent years, there has been growing interest in third-wave CBT approaches that incorporate treatment strategies, such as Acceptance and Commitment Therapy (ACT) and mindfulness. In an open pilot study by Eisenhauer et al., 11 adults with TS received a combination of CBIT and ACT strategies ( Eisenhauer et al. 2025). Analyses showed significantly improved tic severity (as measured by the Yale Global Tic Severity Scale - Total Tic Score: YGTSS-TSS) at post-treatment and at 6- and 12-month follow-ups, indicating the preliminary efficacy of this ACT-enhanced intervention. Reese et al. conducted a pilot RCT ( *N* = 32) comparing two videoconference-delivered group interventions for adults with TS/CTD: mindfulness-based intervention for tics (MBIT) and supportive therapy ( Reese et al. 2024). The results showed a significantly greater reduction in tic severity (YGTSS-TSS) in the MBIT group than in the supportive therapy group at post-treatment, although the superiority was less clear at the 12-month follow-up. These preliminary findings suggest that mindfulness-based interventions may be viable treatment options for individuals with TS/CTD.

A few ERP studies were published in 2024. In a large-scale RCT involving 108 children with TS/CTD, Heijerman-Holtgrefe et al. evaluated a brief, intensive group-based ERP intervention known as the *Tackle Your Tics* program, comparing it to a waitlist control ( Heijerman-Holtgrefe et al. 2024). The intervention included psychoeducation, ERP, separate parent sessions, coping strategy workshops, and relaxation exercises, all delivered over the course of four intensive treatment days. The ERP group showed no superiority to the waiting list in tic-severity improvement (YGTSS-TSS) at post-treatment or at the 3-month follow-up; however, superiority was shown in tic-related impairment and quality of life at the later follow-up. In a long-term follow-up analysis of a previously published RCT, Andrén et al. examined the outcomes of internet-delivered ERP compared to internet-delivered supportive therapy in children with TS/CTD ( *N* = 221) ( Andrén et al. 2024). The initial within-group improvements in tic severity (YGTSS-TTS) were maintained in both treatment groups for up to 12 months post-treatment. Secondary analyses revealed no significant between-group differences in tic severity at any of the time points. However, health-economic evaluations favored ERP overall, indicating greater cost-effectiveness relative to the active control intervention. Although not a clinical trial per se, Rotstein et al. employed the core ERP technique of tic suppression to evaluate a gamified intervention referred to as *XTics* ( Rotstein et al. 2024). In a crossover randomized design involving 35 children with tics, participants played a computerized game incorporating built-in tic triggers and tic suppression exercises. This study compares the effects of immediate and delayed reward contingencies. Overall, the results favored the immediate reward condition, suggesting that combining gamified tic-triggering tasks with immediate in-game reinforcement may be a promising strategy to enhance the efficacy of traditional treatments for TS/CTD, such as ERP.

Another type of BT for TS/CTD is *Cognitive Psychophysiological treatment* (CoPs), which was developed by O’Connor et al. (2016). Unlike CBIT, which focuses on inhibiting tics once triggered, CoPs aim to modify underlying cognitive, behavioral, and psychophysiological processes to prevent tics from being triggered altogether. In an RCT by Leclerc et al., CoPs were compared to CBIT in a sample of 98 children and adults with TS/CTD ( Leclerc et al. 2024). Both treatments showed clinically meaningful within-group improvements in tic severity (YGTSS-TTS) that were maintained up to the 41-week follow-up. However, CoPs did not demonstrate superiority over CBIT. In a study published by the same research group, the influence of ADHD symptomatology on outcomes in CoPs was examined in a sample of 55 adults with TS ( Mazur-Lainé et al. 2024). Using data from a prior open trial, participants were categorized into high- and low-ADHD symptom groups. The analysis revealed no significant differences in tic severity improvement (YGTSS-TTS) between the two groups post-treatment. These findings suggest that BT may not require specific adaptations in individuals with TS and comorbid ADHD.

Recent studies have also compared the efficacy of BT with that of pharmacological treatments. In a retrospective cohort study of 136 children, Wang et al. compared a treatment combining general cognitive-behavioral (CBT) principles with TS-specific HRT with conventional therapy, defined as the use of clonidine transdermal patches ( Wang et al. 2024b). At the 24-week follow-up, the CBT/HRT group showed a significantly greater reduction in tic severity (YGTSS-TTS) than the clonidine group. The limitations of this study include the lack of a randomized, prospesalehctive design. In 2024, van de Griendt et al. conducted an RCT comparing ERP with risperidone in a mixed sample of 30 children and adults with TS ( van de Griendt et al. 2024). Owing to recruitment challenges, only 30 of the planned 80 participants were enrolled, necessitating the use of Bayesian statistical methods. The results indicated comparable effects between the two interventions at the 12-week mark, with a slight advantage for ERP at the 24- and 52-week follow-up. Adverse effects were more common in the risperidone group during the initial treatment phase. Notably, recruitment difficulties were partly attributed to the participants’ reluctance to be randomized to the medication arm.

Future research priorities for BT in TS/CTD were explored by Conelea et al. through a collaborative research planning project involving patients, parents, clinicians, researchers, stakeholder organizations, and other key contributors ( Conelea et al. 2024). This multistage initiative identified several high-priority domains, including improving treatment accessibility and outcomes, optimizing BT within a broader model of care, and expanding outcome measures to include areas beyond tic severity.

**Pharmacological studies**

Many patients with TS have both ADHD and depression, OCD, or an anxiety disorder; therefore, combining a stimulant with a SSRI is common practice. Lee et al. provided evidence from a huge claims database that combining an SSRI with methylphenidate is safe and reduces the risk of headache ( Lee et al. 2024).

For a glimpse into the future, an interesting overview of current therapeutical trials (both pharmacological and non-pharmacological) was provided by Häge et al. (2024).

Pringsheim and Martino offer practical guidelines for the use of botulinum toxin in the treatment of tics, based on the Calgary Adult Tic Registry ( Pringsheim and Martino 2025). Interestingly, botulinum toxin was the most used medication for tics, applied in 32 out of 95 participants (34%). The most common motor tics used were blinking, head turning, and shoulder raising.

**Neurosurgery**

The conceptual framework of deep brain stimulation (DBS) in TS and tic disorders is shifting progressively from structure-centric to network-based approaches. This paradigm allows for reconciliation of the clinical efficacy observed across diverse DBS targets by considering their involvement in overlapping functional networks. Beyond its therapeutic value, this evolving view of DBS also provides crucial insights into the pathophysiology of TS through the lens of brain connectivity.

In a landmark study that included patients with TS, Parkinson disease, dystonia, and OCD, Hollunder et al. (2024) used subthalamic DBS as a means to map dysfunctional neural circuits, introducing the concept of the “dysfunctome.” In TS patients specifically, tic reduction is specifically associated with enhanced connectivity between the stimulation site and both the primary motor cortex and supplementary motor area (SMA).

Building on this network-based approach, Baldermann et al. (2024) conducted an elegant study involving 37 patients treated with thalamic DBS complemented by a cohort of individuals with tic-inducing brain lesions. Their findings revealed that stronger connectivity between DBS targets and regions within the cingulo-opercular and somato-cognitive action networks, including the insula, dorsal anterior cingulate cortex, SMA, and supramarginal gyrus, were predictive of better tic reduction. Moreover, the connectivity profiles of tic-inducing lesions overlapped significantly with these networks, further supporting their relevance in the pathophysiology of TS and reinforcing the value of a network-centric model.

Another promising frontier in DBS is the development of responsive and closed-loop stimulation systems. In a pivotal study, Okun et al. (2024) investigated this approach in ten patients with refractory TS, targeting the centromedian nucleus (CM) of the thalamus and implanting electrodes in the primary motor cortex (M1). They identified tic-related neural signatures in both CM and M1 regions, demonstrating their potential utility as biomarkers for responsive DBS. The widespread availability of a new generation of stimulators with enhanced recording capacity may facilitate the development and implementation of responsive DBS for TS in the near future.

Finally, Gao et al. (2024) conducted a large retrospective cohort study involving 102 patients with TS undergoing pallidal DBS, and compared the outcomes between pediatric and adult populations. Both groups showed progressive clinical improvement over time, with children demonstrating greater reductions in YGTSS scores at the 60-month follow-up (70% vs. 56% in adults). Importantly, no major safety concerns have been reported.

### Tics, family and society

Using data from the randomized controlled’ORBIT’ trial (Online Remote Behavioural Intervention for Tics) ( Hollis et al. 2023), Hall et al. convincingly show that evidence-based online therapy such as ORBIT could save the National Health Service up to £1 million per year, especially by reducing costs of health service use and school absenteeism ( Hall et al. 2024)

Yang et al. examined the psychometric properties of the Chinese version of the Gilles de la Tourette syndrome-quality of life scale (GTS-QOL) for children and adolescents in 1,121 children with TS and found good reliability and validity ( Yang et al. 2024).

Hu et al. investigated parental perspectives on tic disorders in China ( Hu et al. 2024b). The following five themes emerged as most important: physical problems, parenting and education problems, mental problems, bad habits, and neurological problems. TS is frequently related to repetitive searches for medical help owing to insufficient awareness. In addition, parents reported feeling guilty due to poor parenting styles. In some cases, tics is attributed to the bad habits of children.

A focused group of adults with TS explored which topics are important to patients and should be included in the tic registry ( Isaacs et al. 2024c). Various research priorities were identified, such as developing more effective treatments for tics, identifying risk factors for tic persistence into adulthood, elucidating the interaction between TS symptoms and women’s health, clarifying the relationships between TS and other mental and physical health disorders, and addressing day-to-day living issues. Important were also practical issues such as the availability of a wide range of visit times or telehealth options.

Dy-Hollins et al. (2024b) investigated racial and ethnic disparities in a US-based population. Overall, 88% of participants were non-Hispanic whites. Tic onset and age at diagnosis were significantly later in the non-White group.

A systematic review of the public health needs of patients with tics was conducted by Bitsko et al. (2025). While no limitations to healthcare were identified in the group of individuals, children with tics were overall in greater need of specialist care, especially mental health.

Mahajan et al. (2024) examined gender representation in publications dedicated to TS ( Mahajan et al. 2024). After analyzing 1052 publications, it was determined that in 54.8% of all articles, the first authors were female. There was a significant association between sex ratios and the country of publication. Prediction modelling indicated that female participation in TS publications is expected to rise to approximately 60% by the year 2027.

## Conclusions

A few trends emerged in 2024 and might inform future research in the upcoming years. Examples include the classification of tic disorders, given that the DSM-5 was published in 2013, and that there is increased questioning by health care professionals on the difference, or lack thereof, between motor and phonic tics, and between provisional and chronic tic disorders. In addition, learning from the FTLB epidemic, the age of tic onset as diagnostic criteria deserves reconsideration. With regard to FTLB, the wave seems to abate, but confirmatory studies of risk and stress factors consolidate initial clinical intuitions regarding psychological stress factors. Interestingly, in several 2024 publications, it appears that gender minorities are overrepresented in individuals with FTLB, although the cause of this association is unclear.

One interesting finding concerned hyperlearning in TS, suggesting that individuals with tics can outperform healthy controls in certain areas, reminiscent of the classic study on cognitive control published almost two decades ago ( Jackson et al. 2007). There continues to be interest in modulators of tic severity and comorbidities, especially sleep, but also nutrition, an area that deserves further study and is often the focus of interest of parents of children with tics.

Regarding pharmacotherapy, little news appeared in 2024, as everyone awaited the results of the phase III ecopipam trial. However, in the world of behavior therapies, third wave approaches as well as gamifying strategies have emerged, showing that this field is dynamic. Finally, various brain stimulation techniques have been investigated. Closed-loop DBS is an interesting approach that may provide additional improvement compared to standard continuous stimulation modes, but further controlled studies are needed to confirm or refute this hypothesis.

## Ethical considerations

Ethical approval and consent were not required.

## Data Availability

No data are associated with this article.
