# Supplementary material for: Tourette syndrome research highlights from 2024
Source: F1000Res. 2025 Nov 7;14:602. Originally published 2025 Jun 19. [Version 3] doi: 10.12688/f1000research.164800.3 (PMC12547383; doi:10.12688/f1000research.164800.3)
Supplement: Peer review history

# Reviewer report: Reviewer response for version 2

**DOI:** 10.5256/f1000research.187912.r417887

**Published:** 2025-10-22

**Authors:** Lorena Fernández de la Cruz

**Affiliations:** 1 Centre for Psychiatry Research, Department of Clinical Neuroscience, Karolinska Institutet, Stockholm, Sweden

Thank you for inviting me to review this year’s Tourette’s syndrome (TS) research highlights piece. The task of keeping the Tourette’s community up to date is commendable and much appreciated by many of us.

 The scoping narrative review uses PubMed and PubMed Central, as well as recommended articles and selected abstracts from conferences, as the sources to identify the most relevant articles in the field of TS in 2024. The results of the search are presented and summarized in different sections and subsections. This structure feels appropriate and reads well. Based on my own knowledge, I did not notice any obvious omissions of further studies that could have been included. To me, the article represents a good addition to the TS literature that will be helpful to researchers, clinicians, and any other individuals interested in the field. 

 I have a few suggestions for improvement, mainly focused on wording issues and typos. Please see below:

  

- Please check the following sentence in the Methods section. The last bit of it (“On 16 January 2025, 275 citations”) should probably be integrated with the previous clause or reworded in some way: “We searched PubMed using the search strategy (“Tic Disorders”[MeSH] OR Tourette) NOT ((Tourette[AU] OR Tourette[COIS]) NOT (“Tic Disorders”[MeSH] OR Tourette [tiab])) AND 2024 [PDAT] NOT 1800:2023[PDAT]. On 16 January 2025, 275 citations.”

 

- Verbs are missing in the following sentence of the second paragraph of the Definition and phenomenology sentence: “Head/neck tics WERE associated with simple phonic tics, whereas shoulder and trunk tics WERE associated with complex motor tics.” 

 

- Last sentence of the same section: “Participants with coprophenomena had more severe tics and lower scores for global functioning (TS+copro median = 51, TS−copro = 60), family functioning, and quality of life than did participants without coprophenomena.” I would be inclined to remove the parenthesis presenting the median scores given that the sentence already reports the result, and other outcomes mentioned do not include numerical results. Or alternatively, explain what those scores are. Are they referred to a specific measure of global functioning?

 

- It would be good to briefly mention how the diagnosis of TS was assessed in the only study mentioned under the Epidemiology section. Additionally, in this small paragraph, there probably is an “and” missing before the clause “found a prevalence of 0.23%”.

 

- Environmental risk factors section: “Twenty-four patients with obsessive-compulsive tic disorder were included in a longitudinal study lasting 15 months in Italy (Lamanna et al. 2024).” Please define what it is understood here by “obsessive-compulsive tic disorder” given that this nomenclature is not used (as such) in the main classificatory systems.

 

- There are acronyms that are defined more than once (e.g., ADHD, OCD, TS) and there are other times where an acronym has been previously defined but later on the name in full is used. Please check throughout that an acronym is defined the first time is mentioned and that only the acronym is used from then on.

Is the review written in accessible language?

Yes

Are all factual statements correct and adequately supported by citations?

Yes

Are the conclusions drawn appropriate in the context of the current research literature?

Yes

Is the topic of the review discussed comprehensively in the context of the current literature?

Yes

Reviewer Expertise:

Obsessive-compulsive and related disorders, tic diorders, epidemiology, psychological treatments

I confirm that I have read this submission and believe that I have an appropriate level of expertise to confirm that it is of an acceptable scientific standard.

# Response

**Published:** 2025-10-23

**Authors:** Andreas Hartmann

**Affiliations:** Pitié-Salpêtrière Hospital, Paris, France

We thank Dr Fernández de la Cruz for carefully reading our manuscript and making suggestions for its improvement.

- Please check the following sentence in the Methods section. The last bit of it (“On 16 January 2025, 275 citations”) should probably be integrated with the previous clause or reworded in some way: “We searched PubMed using the search strategy (“Tic Disorders”[MeSH] OR Tourette) NOT ((Tourette[AU] OR Tourette[COIS]) NOT (“Tic Disorders”[MeSH] OR Tourette [tiab])) AND 2024 [PDAT] NOT 1800:2023[PDAT]. On 16 January 2025, 275 citations.”

**Response: **Thank you for the suggestion, the passage was modified accordingly.

- Verbs are missing in the following sentence of the second paragraph of the Definition and phenomenology sentence: “Head/neck tics WERE associated with simple phonic tics, whereas shoulder and trunk tics WERE associated with complex motor tics.” 

**Response: **Thank you for the suggestion, the passage was modified accordingly.

- Last sentence of the same section: “Participants with coprophenomena had more severe tics and lower scores for global functioning (TS+copro median = 51, TS−copro = 60), family functioning, and quality of life than did participants without coprophenomena.” I would be inclined to remove the parenthesis presenting the median scores given that the sentence already reports the result, and other outcomes mentioned do not include numerical results. Or alternatively, explain what those scores are. Are they referred to a specific measure of global functioning?

**Response: **Thank you for the suggestion, the parenthesis was removed.

  

- It would be good to briefly mention how the diagnosis of TS was assessed in the only study mentioned under the Epidemiology section. Additionally, in this small paragraph, there probably is an “and” missing before the clause “found a prevalence of 0.23%”.

 
**Response: **Thank you for the suggestion, the passage was modified accordingly.

  

- Environmental risk factors section: “Twenty-four patients with obsessive-compulsive tic disorder were included in a longitudinal study lasting 15 months in Italy (Lamanna et al. 2024).” Please define what it is understood here by “obsessive-compulsive tic disorder” given that this nomenclature is not used (as such) in the main classificatory systems.

**Response: **Thank you for the suggestion, the passage was modified accordingly.

- There are acronyms that are defined more than once (e.g., ADHD, OCD, TS) and there are other times where an acronym has been previously defined but later on the name in full is used. Please check throughout that an acronym is defined the first time is mentioned and that only the acronym is used from then on.

**Response: **Thank you for the suggestion, the passage was modified accordingly.

# Reviewer report: Reviewer response for version 1

**DOI:** 10.5256/f1000research.181375.r393490

**Published:** 2025-08-09

**Authors:** I-Ching I-Ching Chou

**Affiliations:** 1 China Medical University, Taichung, Taiwan

The article titled “Tourette syndrome research highlights from 2024” provides an extensive and thoughtfully curated annual summary of key advances in Tourette syndrome (TS) research. Compiled by an international team of leading researchers, it spans a wide range of domains: phenomenology, diagnostic criteria, assessment tools, prognosis, comorbidities, functional tic-like behaviors (FTLB), genetics, neuroimaging, pathophysiology, neuropsychology, animal models, and treatment (both behavioral and pharmacological). The review is positioned as part of an ongoing annual series and aims to provide both clinicians and researchers with an accessible synthesis of the most important publications of the year.

**Suggestions for Improvement (Minor):**

- **Clarify inclusion criteria:** While the PubMed search strategy is described, the exact criteria for selecting the “most important or interesting” studies remain subjective. A brief table summarizing selected articles by domain (e.g., genetics, treatment, etc.) with justification could enhance transparency.
- **Improve figure/tables (if any):** The manuscript would benefit from a graphical summary (e.g., timeline or research landscape map) to enhance readability, particularly for trainees or non-specialists.
- **Address potential bias:** While disclosures are thorough, it may be helpful to emphasize how any financial or research conflicts were managed when selecting or interpreting studies.

**Conclusion & Recommendation:**

 This is a well-executed, comprehensive, and valuable synthesis of current TS research. It meets the standards of scientific rigor, clarity, and clinical relevance expected of an annual highlights publication.

Is the review written in accessible language?

Yes

Are all factual statements correct and adequately supported by citations?

Yes

Are the conclusions drawn appropriate in the context of the current research literature?

Yes

Is the topic of the review discussed comprehensively in the context of the current literature?

Yes

Reviewer Expertise:

Pediatric Neurology

I confirm that I have read this submission and believe that I have an appropriate level of expertise to confirm that it is of an acceptable scientific standard.

# Response

**Published:** 2025-09-08

**Authors:** Andreas Hartmann

**Affiliations:** Pitié-Salpêtrière Hospital, Paris, France

- **Clarify inclusion criteria:** While the PubMed search strategy is described, the exact criteria for selecting the “most important or interesting” studies remain subjective. A brief table summarizing selected articles by domain (e.g., genetics, treatment, etc.) with justification could enhance transparency.

Our inclusion criteria have been subjective from the inception (2016) and we cannot see how to objectify these, or if it would even be desirable. This is a multi-authoe effort that pools different areas of subexpertise in the field of Tourette syndrome. We choose articles, as explained in the introduction, based on the potential interest to our readers but fully realize that our choices will always be debatable, and offer apologies to papers we haven’t included even if they certainly have merits. Our main goal is and always has been to show that research in TS is a vibrant and expanding field. Accordingly, the length of this paper has grown substantially over the last decade, and we fear that adding a table summarizing the studies presented in the text section will literally explode the frame we are allowed to use.   

  

- **Improve figure/tables (if any):** The manuscript would benefit from a graphical summary (e.g., timeline or research landscape map) to enhance readability, particularly for trainees or non-specialists.

We are not quite quite sure what the referree means. The timeline is January 1st to December 31st, 2024. Our search strategy, based on publicly available databases, is outlined in the introductory paragraph, and has been the same since 2016.

  

- **Address potential bias:** While disclosures are thorough, it may be helpful to emphasize how any financial or research conflicts were managed when selecting or interpreting studies.

Since we did not present any commercial studies in this review, there are no potential conflicts of interest. Next year, when discussing the results of the Diamond study (Ecopipam) – if published in 2025 – some of the authors will indeed point out that they have been co-PIs. That, however, should not bias the main conclusions of the study and we will certainly not formulate any therapeutic recommendations.

**Conclusion & Recommendation:**

 This is a well-executed, comprehensive, and valuable synthesis of current TS research. It meets the standards of scientific rigor, clarity, and clinical relevance expected of an annual highlights publication.

 We thank the referree for his kind words.

# Reviewer report: Reviewer response for version 1

**DOI:** 10.5256/f1000research.181375.r393484

**Published:** 2025-08-08

**Authors:** Erica L. Greenberg

**Affiliations:** 1 Department of Psychiatry, Harvard Medical School, Boston, MA, USA

Thank you for the opportunity to review this comprehensive manuscript that describes the recent findings on Tourette syndrome during 2024. It was well-written, well-constructed, generally clear and provided practical and thoughtful commentary on the state of the field to date. I really enjoyed reading it and took away a number of important facts! I have a few comments/suggestions that would benefit from further review by the authors: (Of note, there were a handful of spelling/small grammatical errors. I try to note these below, but I may have missed a few). 

**Results:**

 Phenomenology and Natural History

  - Beginning "an analysis of a large TS genetics..." - would help to briefly define lower functional inflexibility

  - Beginning "axial tics" - Im not sure this reflects the text accurately enough given that axial tics were identified in 75% of the population, and that head/neck tics and shoulder/trunk tics had different associations

  - Beginning "Green et al" - could help to add an example in the last sentence regarding which type of medication the authors are referring to... e.g. gabapentin? If not something like gabapentin, not sure what authors of the manuscript would be referring to

  - Beginning "Szejko et al..." would re-write last sentence as not clear what "this" is referring to re detrimental effect on quality of life

  - Beginning "A group from Taiwan..." - I would recommend re-writing this blurb. It would help if the authors of the manuscript described what the scale was examining (beyond just 'care needs', as it's not clear why it was compared to the Pittsburgh sleep scale. (Also it says it has demonstrated correlation with itself, which I presume is a typo?)

  - Beginning "Shappert et al" - would help to mention the characteristics of the participants in the study - youth, adults, how many, for how long were they assessed, etc

  - Beginning "two reports provided the first..." - it says that "remission was predicted by the following factors: disease duration>3 months, moderate/severe tic severity, and comrobid behavioral symptoms" - was that supposed to be the opposite? Non-remission predicted by those? Also not clear what is meant by "the investigator observed tics during a 70% of the long 12-month visits"

  - Beginning "Bootes et al" - would re-write this summary - not sure what they are describing... predictors of impairment *and *self-concept? What does it mean for self-concept to be associated with anxiety disorder etc.

  - Beginning "Prato et al.." I found this description difficult to follow as well. Wasn't clear whether this was comparing groups with TS+ASD vs TS-ASD vs ASD-TS? If not, not clear why premonitory urges were significantly more represented in the ASD group versus the TS group (unless that is a typo and should be sensory phenomena)? 

  - Beginning "Kurvits et al" - would help to define for the reader what a 'noisy voluntary movement' is? And/or what sensorimotor noise is.

  - Beginning "A symptom network..." would recommend re-writing as in general, I found this description difficult to follow; particularly the sentence "core symptom networks include complex tics and tic-related phenomena and touching people and objects."

  - Beginning "Jensen et al..." - would either eliminate the last sentence or adjust to describing it by what the authors of the article think; otherwise it reads as a discussion point by the manuscript's authors. 

  - Beginning "Zoccante et al..." - says "...hypersensitivity were more common in patients with TS" but compared to what? Controls? Those with only ASD or ADHD?

  - Beginning "The frequency of co-occurring somatic diseases..." - would not include ADHD in the list given it doesn't fit with the others (and is neuropsychiatric vs somatic)

  - Beginning "Chen et al..." - where it says "and a higher frequency of psychosocial factors" I believe that should be "psychological factors" given what they are referring to (anxiety and anger)

  - Beginning "Temperament traits in patients..." - missing the word 'was' between harm avoidance and increased.

  - Beginning "A group from Iran" - would re-write as difficult to follow, particularly the first sentence

  - Beginning "Fiche et al..." - would help to clarify what a "private" variant?

  - Beginning "Salehi et al..." would rewrite as difficult to follow what was being assessed and what the conclusions were 

 Pathophysiology:

  - Beginning "In 2024, Takacs et al..." - would help to briefly explain what "statistical learning" represents (compared to rule-based learning). Also to explain what "small-world-like properties" refers to

  - Beginning "A study conducted by Zapparoli et al..." - not sure what is meant by "relief during tic inhibition" as I imagine that inhibiting tics is not associated with relief... and not sure if this is a mi-statement

**Treatment:**

  - Beginning "Several reports have provided..." - perhaps just me but having trouble trying to conceptualize what is meant by a negative pooled effect size for placebo; brief explanation for reader might be helpful

  - Beginning "Current clinical guidelines..." - word "habitat" is written instead of "habit"

  - Beginning "Another type of BT for TS/CTD..." - the authors say "...contrary to our expectations" - this should be changed to 'contrary to the authors' expectations,' since otherwise would be a point for the discussion. 

  - Beginning "Recent studies have also compared..." - where the authors suggest that ERP may be viewed as more acceptable, would change that to either 'the authors suggest that ERP may be viewed as more acceptable' or would clarify that it may be depend on where the recruitment was taking place; for example, if recruitment was at predominantly a medication management program, might have had the opposite finding re not wanting to be randomized to behavioral.

  - Beginning "Pringsheim and Martino..." - not sure why only 95 participants if it's an Adult Tic Registry... is it a new registry? Trying to put in perspective why botox is most represented as not sure if it's a generalizable cohort depending on who was eligible

  - Beginning "In a landmark study..." do the authors mean, "In [TS] patients specifically?"

  - Beginning "Dy-Hollins et al..." - would re-write as appears to be grammatical/cutting-pasting errors; and also want to confirm that "age of diagnosis was *earlier *in non-White group. 

  - Beginning "Mahajan et al..." - would also re-write as difficult to follow/looks like cut/paste errors

**Conclusions:**

  - The sentence(s) beginning "In addition, learning from the FTLB epidemic" through "initial clinical intuitions" is difficult to follow; would recommend re-writing. 

  - Beginning "Regarding pharmacotherapy..." might eliminate the phrase "the field is alive and kicking" given it feels less formal/more colloquial compared to the rest of the manuscript.

Is the review written in accessible language?

Yes

Are all factual statements correct and adequately supported by citations?

Partly

Are the conclusions drawn appropriate in the context of the current research literature?

Yes

Is the topic of the review discussed comprehensively in the context of the current literature?

Yes

Reviewer Expertise:

Tourette syndrome, common comorbidities, child and adolescent psychiatry

I confirm that I have read this submission and believe that I have an appropriate level of expertise to confirm that it is of an acceptable scientific standard, however I have significant reservations, as outlined above.

# Response

**Published:** 2025-09-08

**Authors:** Andreas Hartmann

**Affiliations:** Pitié-Salpêtrière Hospital, Paris, France

**Results:**

**Phenomenology and Natural History**

**- Beginning "an analysis of a large TS genetics..." - would help to briefly define lower functional inflexibility.**

 Done. Functional flexibility = difficulty switching strategies, adapting to new rules, or adjusting to unexpected changes in environment or routine.

**- Beginning "axial tics" - Im not sure this reflects the text accurately enough given that axial tics were identified in 75% of the population, and that head/neck tics and shoulder/trunk tics had different associations. -**

 Done, the text has been expanded.

**- Beginning "Green et al" - could help to add an example in the last sentence regarding which type of medication the authors are referring to... e.g. gabapentin? If not something like gabapentin, not sure what authors of the manuscript would be referring to**

 Done. It is not gabapentin but botulinum toxin and cannabis/cannabinoids.

**- Beginning "Szejko et al..." would re-write last sentence as not clear what "this" is referring to re detrimental effect on quality of life**

 Done.

**- Beginning "A group from Taiwan..." - I would recommend re-writing this blurb. It would help if the authors of the manuscript described what the scale was examining (beyond just 'care needs', as it's not clear why it was compared to the Pittsburgh sleep scale. (Also it says it has demonstrated correlation with itself, which I presume is a typo?)**

 Done. We have revised the paragraph to clarify what the Care Needs Scale for Parents of Children with TS (CNS-PCTS) assesses, and why it was compared to the Pittsburgh Sleep Quality Index. We also corrected the phrasing regarding correlation, as the original wording was unclear and inadvertently suggested the scale correlated with itself. The revised text now emphasizes that the CNS-PCTS was designed to measure the multidimensional care needs of parents (including emotional support, information needs, and practical assistance) and that its correlation with the Pittsburgh Sleep Quality Index supports its construct validity, given the known relationship between caregiver burden and sleep quality « This instrument was designed to assess the multidimensional needs of parents, including emotional support, informational needs, and practical aspects of caregiving. The scale demonstrated excellent content and discriminant validity, and showed significant correlations with the Pittsburgh Sleep Quality Index, supporting its construct validity in relation to caregiver well-being. »

**- Beginning "Shappert et al" - would help to mention the characteristics of the participants in the study - youth, adults, how many, for how long were they assessed, etc**

 Done. We have revised the paragraph to include details on the study population, including age group, sample size, and assessment duration. This additional information clarifies the context and applicability of the automated video-based diagnostic approach « Schappert et al. (2024) evaluated an automated video-based approach to diagnosing Tourette syndrome in a cohort of 42 adult participants, analyzing a total of 107 videos. Using automated classification of tic frequency and temporal clustering, the system achieved a balanced accuracy of 87.9% for tic quantity and 90.2% for tic clusters. When approximately 25% of the lower-confidence cases were reviewed by experts, the overall diagnostic accuracy exceeded 95% » ( Schappert et al. 2024).

**- Beginning "two reports provided the first..." - it says that "remission was predicted by the following factors: disease duration>3 months, moderate/severe tic severity, and comrobid behavioral symptoms" - was that supposed to be the opposite? Non-remission predicted by those? Also not clear what is meant by "the investigator observed tics during a 70% of the long 12-month visits"**

 Done, and we thank Dr. Greenberg for this important clarification. She is correct that in the Liu et al. study, non-remission was associated with disease duration >3 months, moderate/severe tic severity, and comorbid behavioral symptoms. We have revised the text accordingly to avoid misinterpretation.

 Regarding the Grossen et al. report, we agree that our original phrasing was unclear. What the authors found was that at the 12-month assessment, tics were directly observed by the clinician in 70% of visits. However, when additional observation by video was performed while the child was alone in the room, tics were detected in all but one participant. We have rephrased this section to make the meaning clear: “Two reports provided the first large prospective follow-up studies on provisional tic disorder (PTD). Liu et al. studied prognostic factors in children with PTD. After 1 year of follow-up, clinical remission was observed in 30% of cases. Persistence of tics (non-remission) was associated with longer disease duration (>3 months), greater tic severity, and comorbid behavioral symptoms (Liu et al., 2024). A report from the US used a more intensive assessment 12 months after tic onset (Grossen et al., 2024). At that visit, tics were observed directly during 70% of standard clinical encounters. However, when the children were additionally monitored on video while alone in the room, tics were present in all but one of 79 cases. This result is consistent with prior clinical experience, where the absence of tics during routine examination is common. The authors concluded that PTD is much more similar to TS than different, and they argue that the traditional but arbitrary separation of provisional from chronic tic disorders should be reconsidered”.

**- Beginning "Bootes et al" - would re-write this summary - not sure what they are describing... predictors of impairment *and *self-concept? What does it mean for self-concept to be associated with anxiety disorder etc.**

 We thank Dr. Greenberg for pointing out that our description of Bootes et al. (2024) was unclear. In their study, the authors examined predictors of both functional impairment and self-concept in children and adolescents with persistent tic disorder. They found that poorer self-concept (i.e., more negative views of the self) was linked to comorbid anxiety disorder, depression, greater tic severity and complexity, and worse quality of life. We have revised this section to make the findings clearer and to specify the direction of associations : « Bootes et al. (2024) investigated predictors of functional impairment and self-concept in children and adolescents with persistent tic disorder. They found that poorer self-concept (a more negative view of oneself) was associated with comorbid anxiety and depression, greater tic severity and complexity, and lower quality of life. »

**- Beginning "Prato et al.." I found this description difficult to follow as well. Wasn't clear whether this was comparing groups with TS+ASD vs TS-ASD vs ASD-TS? If not, not clear why premonitory urges were significantly more represented in the ASD group versus the TS group (unless that is a typo and should be sensory phenomena)?**

 We thank Dr. Greenberg for noting that our description of Prato et al. (2024) was confusing. The study compared three groups: children with Tourette syndrome (TS), children with autism spectrum disorder (ASD), and healthy controls. Using the University of São Paulo Sensory Phenomena Scale (USP-SPS), the authors found that sensory phenomena were significantly more represented in the ASD group compared with the TS group (with the exception of sound-related “just-right” perceptions and the feeling of energy to be released). ASD participants also showed higher mean severity scores across all USP-SPS domains compared with both TS patients and controls. The severity of sensory phenomena was positively correlated with OCD and anxiety severity, and in the ASD group specifically, with ADOS scores and inversely with IQ. We have revised the text to clarify that the finding concerned sensory phenomena broadly, not only premonitory urges, and to specify the groups compared : « Prato et al. (2024) investigated sensory phenomena in children with Tourette syndrome (TS) and autism spectrum disorder (ASD) using the University of São Paulo Sensory Phenomena Scale (USP-SPS). They found that sensory phenomena were more prevalent and severe in the ASD group compared with the TS group, except for sound-related “just-right” perceptions and the sensation of energy to be released. On average, ASD participants scored higher than both TS patients and healthy controls across all USP-SPS severity domains. Greater severity of sensory phenomena was associated with more severe obsessive-compulsive and anxiety symptoms; in the ASD group, it was also positively correlated with ADOS total scores and negatively correlated with IQ. »

**- Beginning "Kurvits et al" - would help to define for the reader what a 'noisy voluntary movement' is? And/or what sensorimotor noise is.**

 We thank Dr. Greenberg for this helpful suggestion. In Kurvits et al. (2024), *“noisy voluntary movements”* refers to voluntary actions that show greater variability or inconsistency in their execution. This variability is understood as increased *sensorimotor noise*, i.e., random fluctuations in the neural signals that guide movement. We have revised the text to briefly define these terms for clarity : « Kurvits et al. (2024) investigated rapid compensation for “noisy” voluntary movements in individuals with tic disorders, based on the premise that tics and premonitory movements may reflect increased sensorimotor noise (random variability in the neural signals controlling movement). They found that patients with tics produced more variable voluntary movements than controls, but were able to rapidly adjust their performance according to current task demands. »

**- Beginning "A symptom network..." would recommend re-writing as in general, I found this description difficult to follow; particularly the sentence "core symptom networks include complex tics and tic-related phenomena and touching people and objects."**

 We thank Dr. Greenberg for highlighting that our description of Forlim et al. (2024) was difficult to follow. The study applied network analysis to identify *core symptoms*—that is, symptoms that are highly connected to others and may drive overall symptom severity—in children and adults with chronic tic disorders. We have rewritten this section to more clearly explain which symptoms emerged as central in the network and how they differed by age group : « Forlim et al. (2024) conducted a symptom network analysis in a large sample of children and adults with chronic tic disorders. In the overall network, complex motor and vocal tics, as well as behaviors such as touching people or objects, emerged as central (highly connected) symptoms. In children, attention-deficit/hyperactivity disorder (ADHD) symptoms were among the most influential nodes, whereas in adults, obsessive-compulsive disorder (OCD) symptoms were more central. Self-injurious behaviors were peripheral in children but constituted a core feature in the adult network. The analysis also revealed strong interconnections between complex tics, echolalia, and echopraxia ».

**- Beginning "Jensen et al..." - would either eliminate the last sentence or adjust to describing it by what the authors of the article think; otherwise it reads as a discussion point by the manuscript's authors.**

 Done. The last sentence was eliminated.

**- Beginning "Zoccante et al..." - says "...hypersensitivity were more common in patients with TS" but compared to what? Controls? Those with only ASD or ADHD?**

 Done. Compared to (healthy) controls, as mentioned. the ASD and ADHD groups also showed differences compared to controls but we simply mention the TS group here.

**- Beginning "The frequency of co-occurring somatic diseases..." - would not include ADHD in the list given it doesn't fit with the others (and is neuropsychiatric vs somatic)**

 Done. We removed the ADHD (although the authors of the manuscript would maybe argue that ADHD is a somatic disease after all).

**- Beginning "Chen et al..." - where it says "and a higher frequency of psychosocial factors" I believe that should be "psychological factors" given what they are referring to (anxiety and anger)**

 Done, and agreed.

**- Beginning "Temperament traits in patients..." - missing the word 'was' between harm avoidance and increased.**

 Done.

**- Beginning "A group from Iran" - would re-write as difficult to follow, particularly the first sentence**

 We thank Dr. Greenberg for pointing out that our description of Esmaeilzadeh et al. (2024) was confusing. The study compared three groups: (1) patients with both tic disorders and allergic rhinitis (AR), (2) patients with tic disorders without AR, and (3) patients with AR without tics. We have revised the text to make the design and results clearer : « Esmaeilzadeh et al. (2024) conducted a case–control study in Iran comparing three groups: patients with both tic disorders and allergic rhinitis (AR), patients with tic disorders without AR, and patients with AR without tics. They found that vocal tics were more frequent among patients with both tics and AR, and these individuals also experienced more days per week with AR symptoms. The most common tic diagnosis in the sample was provisional tic disorder. These findings suggest a possible interaction between allergic symptoms and tic expression, highlighting the need to consider comorbid allergic conditions in the assessment and management of tic disorders ».

**- Beginning "Fiche et al..." - would help to clarify what a "private" variant?**

 Done. We updated the paragraph providing a clear explanation of the “private” variants.

**- Beginning "Salehi et al..." would rewrite as difficult to follow what was being assessed and what the conclusions were**

 Done. We extended the paragraph, hoping it is now easier to understand.

 Pathophysiology:

**- Beginning "In 2024, Takacs et al..." - would help to briefly explain what "statistical learning" represents (compared to rule-based learning). Also to explain what "small-world-like properties" refers to**

 Done. We included definitions of statistical learning, rule-based learning, and small-world-like properties.

**- Beginning "A study conducted by Zapparoli et al..." - not sure what is meant by "relief during tic inhibition" as I imagine that inhibiting tics is not associated with relief... and not sure if this is a mi-statement**

 Yes, the word “relief” in that sentence was a typo. We removed it.

**Treatment:**

**- Beginning "Several reports have provided..." - perhaps just me but having trouble trying to conceptualize what is meant by a negative pooled effect size for placebo; brief explanation for reader might be helpful**

 Done. We have added a sentence explaining the concept of pooled affect size.

**- Beginning "Current clinical guidelines..." - word "habitat" is written instead of "habit"**

 Done.

**- Beginning "Another type of BT for TS/CTD..." - the authors say "...contrary to our expectations" - this should be changed to 'contrary to the authors' expectations,' since otherwise would be a point for the discussion.**

 Done.  We removed the part about expectations, so it reads now: « However, CoPs did not demonstrate superiority over CBIT. »

**- Beginning "Recent studies have also compared..." - where the authors suggest that ERP may be viewed as more acceptable, would change that to either 'the authors suggest that ERP may be viewed as more acceptable' or would clarify that it may be depend on where the recruitment was taking place; for example, if recruitment was at predominantly a medication management program, might have had the opposite finding re not wanting to be randomized to behavioral.**

 Done.  We removed the added flavour of discussion/interpretation by removing the last part, so it now reads: « Notably, recruitment difficulties were partly attributed to the participants’ reluctance to be randomized to the medication arm. »

**- Beginning "Pringsheim and Martino..." - not sure why only 95 participants if it's an Adult Tic Registry... is it a new registry? Trying to put in perspective why botox is most represented as not sure if it's a generalizable cohort depending on who was eligible**

 Yes, it is a (rather) new registry and this was the number of patients available at the time of analysis (it has grown since). We simply convey the conclusions of the authors: everybody is free to read to the paper and disagree with them, for instance regarding the possible generalization of their findings. Personally, we believe that BTX is an underutilized resource in the treatment of tics and therefore feel this study deserves to be mentioned. Dr. Martino also presented this at the ESSTS meeting in Varese, June 2024.

**- Beginning "In a landmark study..." do the authors mean, "In [TS] patients specifically?"**

 Done, and yes, TS was “swallowed”.

**- Beginning "Dy-Hollins et al..." - would re-write as appears to be grammatical/cutting-pasting errors; and also want to confirm that "age of diagnosis was *earlier *in non-White group.**

 Done. And Dr. Greenberg is correct, tic onset and tic diagnosis occurred later in the non-White group.

**- Beginning "Mahajan et al..." - would also re-write as difficult to follow/looks like cut/paste errors**

 Done. The sentence was re-written.

**Conclusions:**

**- The sentence(s) beginning "In addition, learning from the FTLB epidemic" through "initial clinical intuitions" is difficult to follow; would recommend re-writing.**

 Done. The sentence was re-written.

**- Beginning "Regarding pharmacotherapy..." might eliminate the phrase "the field is alive and kicking" given it feels less formal/more colloquial compared to the rest of the manuscript.**

 Done. « Alive and kicking » was replaced by « dynamic ».
